# Dornase alfa in Cystic Fibrosis: indications, comparative studies and effects on lung clearance index

**DOI:** 10.1186/s13052-022-01331-5

**Published:** 2022-08-04

**Authors:** Vito Terlizzi, Chiara Castellani, Giovanni Taccetti, Beatrice Ferrari

**Affiliations:** 1 Department of Paediatric Medicine, Meyer Children’s Hospital, Cystic Fibrosis Regional Reference Center, Viale Gaetano Pieraccini 24, 50139 Florence, Italy; 2 Meyer Children’s Hospital, Rehabilitation Unit, Florence, Italy

**Keywords:** Mucolytic agents, LCI, Children, Lung function

## Abstract

Cystic fibrosis (CF) is the most common inherited disease in Caucasian populations, affecting around 50,000 patients in Europe and 30,000 in United States. A mutation in CF trans-membrane conductance regulator (*CFTR*) gene changes a protein (a regulated chloride channel), which is expressed in many tissues. Defective *CFTR* results in reduced chloride secretion and an overage absorption of sodium across the epithelia, leading to thickened secretions in organs such as pancreas and lung. Gradually, there have been considerable improvements in the survival of people with CF, thanks to substantial changes in specialized CF care and the discovery of new *CFTR* modulators drugs. Nevertheless, lung disease remains the most common cause of death. For these reasons improvement of sputum clearance is a major therapeutic aim in CF. So far, symptomatic mucolytic therapy is mainly based on inhalation of dornase alfa, hypertonic saline or mannitol, in combination with physiotherapy. The major component of mucus in CF is pus including viscous material such as polymerized DNA derived from degraded neutrophils. Dornase alfa cleaves the DNA released from the neutrophils and reduces mucous viscosity, and further prevent airway infections and damage to the lung parenchyma. In this review we will summarize the current knowledge on dornase alfa in the treatment of CF lung disease, especially highlighting the positive effect on lung clearance index, a sensitive measure of ventilation inhomogeneity.

## Introduction

Cystic fibrosis (CF) is a life-limiting autosomal recessive disorder due to variants in the CF Transmembrane Conductance Regulator (*CFTR*) gene. *CFTR* protein is responsible for chloride ion transport across apical epithelial cells in tissues of the airway, intestine, pancreas, kidney, sweat gland, and male reproductive tract [1]. Furthermore, it has many other regulatory roles, such as bicarbonate secretion, which regulates the pH of airway surface liquid, and inhibition of the epithelial sodium channel, which has an important role in the hydration of secretions and mucins [2].

To date, 360 *CFTR* variants are known to be CF-causing (https://cftr2.org/). CF-causing variants are classified into 6 categories, according to their impact on the production, trafficking, functioning or stability of the *CFTR* channel. Variants belonging to classes I, II and III usually result in little to no *CFTR* activity, leading to severe clinical outcomes, whilst variants from classes IV, V and VI allow significant residual *CFTR* function leading to milder phenotypes [3]. Although the pancreatic status is closely related to the *CFTR* genotype [4], there is a wide clinical heterogeneity in CF patients, even between those carrying the same *CFTR* genotype or between siblings with CF [5], partly explained by environmental factors or by the relevant role of genes other than *CFTR*, defined as “modifying genes” [6–8].

There has been a steady increase in newborn screening (NBS) for CF across Europe over the past ten years [9–11]. CF NBS, when associated with early treatment, limits lung damage in childhood, has a beneficial effect on clinical outcomes, reduces the burden of care for families, and may improve survival [1, 11]. Undesired consequences of NBS for CF include the identification of carrier status [12] and the emergence of a cohort of infants with positive NBS test results but an inconclusive diagnosis [13–15]. Sweat test according to Gibson and Cooke’s method [16] is still the diagnostic gold standard for CF diagnosis. In individuals presenting with a positive NBS, clinical features consistent with CF, or a positive family history, a diagnosis of CF can be made if the sweat chloride value is ≥ 60 mmol/L [17]. The CF phenotype is characterised by lung disease (bronchiectasis with persistent airway-based infection and inflammation), exocrine pancreatic insufficiency associated with nutrient malabsorption contributing to malnutriton, impaired growth, hepatobiliary manifestations, and male infertility [1]. On the other hand, the diagnosis of CFTR-related disorder has been defined as a mono-symptomatic clinical entity, such as in presence of congenital bilateral absence of the vas deferens, pancreatitis or bronchiectasis at computed tomography scan, associated with *CFTR* dysfunction that does not fulfil the diagnostic criteria for CF [18].

Today *CFTR* modulator therapies targeting the basic molecular defect in CF have been developed for specific *CFTR* variants and are associated with improved health outcomes, including improved respiratory function and nutritional status, and enhanced quality of life. They are bringing hope for patients and progress in the development of such drugs has been substantial over the past decade [19–22].

Over time, the survival of patients with CF significantly improved; nevertheless, lung disease remains the most common cause of death and symptomatic mucolytic therapy drugs remain crucial for reducing secretion build-up, preventing infections and slowing lung damage. Mucolytic agents break down the gelatinous structure of mucus and therefore decrease its elasticity and viscosity, reducing the pulmonary exacerbation frequency and to improve and stabilize lung function. However, high quality studies comparing these mucolytic drugs are still lacking, and the individual experiences of patients and caregivers explain the high variability of their use globally [1].

In this review we summarize the current knowledge on dornase alfa (DNAse) in the treatment of CF lung disease, carrying out a systematic review of peer-reviewed literature using Medline/ PubMed, Cochrane, Google Scholar or the online database “Cystic Fibrosis DataBase” (CFDB; https://www.cfdb.eu/en/topics/detail/code/091). Furthermore, we report the positive effects of this drug on lung ventilation homogeneity measured as lung clearance index (LCI).

## Dornase alfa

### Generality

DNAse (Pulmozyme®) is a highly purified solution of recombinant human deoxyribonuclease. The enzyme is naturally present in human tissues but as a drug it is produced industrially through the bacterial synthesis (according to the “recombinant DNA2 technique) and, administered by inhalation, it fragments the DNA molecules, reducing mucus viscosity in the lungs and promoting improved clearance of secretions [23–25]. On the other hand, medications such as hypertonic saline and mannitol are osmotically active and improve mucociliary clearance by rehydrating the airway surface liquid.

The first laboratory evidence of the ability of DNAse to reduce the viscosity of the mucus date back to 1950s when a bovine compound had been used [26]. In 1990 DNAse was produced and since 1992 it has been used as a mucolytic to treat people with CF.

Today DNAse is the only mucus degrading agent that has proven efficacy in CF [27] and it is commonly used in the treatment and management of CF in conjunction with standard therapies. Furthermore, there are also several studies demonstrating the use of DNAse in non-CF patients but this is not the topic of our paper.

### Indications and timing of DNAse inhalation for cystic fibrosis patients

DNAse was recommended by CF Foundation (CFF) Guidelines as a standard of treatment for children 6 years and above with mild to severe lung disease [28] and in the standards of care of the European CF Society [27]. Nevertheless, the use of DNAse is very different among countries. In the US, the 2020 CFF Patient Registry reports that 91.5% of patients > 6 years of age used DNAse [29]. In Europe, this range is very wide, just over 40% in Italy [30].

The recommended dose for use in most people with CF is 2.5 mg (in one single-use ampoule) inhaled once daily using a recommended nebuliser [25].

Fuchs et al. compared the effect of DNAse on Forced Expiratory Volume in the 1st second (FEV_1_) in two groups of patients who used the drug once or twice daily, respectively. They did not show significant differences [31]. On the other hand, Suri et al. evaluated the effect of DNAse on FEV_1_ in two groups of children (N. 48) taking the drug for 12 weeks once daily or every other day. No significant differences were shown given the difference in FEV_1_ increase of 2% [32].

Furthermore, Dentice R and Elkins M evaluated whether the timing of DNAse inhalation, in relation to airway clearance techniques or morning versus evening inhalation, had an impact on objective and subjective measures of clinical efficacy in people with CF. Although the evidence derived from a small number of participants (n. 98 from 4 trials), no improvement resulted from inhalation of DNAse after airway clearance techniques. So the authors concluded that the timing of DNAse inhalation could be largely based on pragmatic reasons or individual preference with respect to the time of airway clearance and time of day [33].

### Comparative studies between DNAse and placebo or other drugs

There were 15 trials comparing DNAse vs placebo [25]. The treatment duration ranged from 6 days to 3 years with variables wash-out periods. The dose of DNAse used was always 2.5 mg, once or twice a day.

A multicenter, double-blind, randomized study evaluated the effect of DNAse used for 12 weeks in 320 CF patients with severe lung disease. The two groups were comparable for age, height and weight. The treated group with DNAse showed at the end of the treatment period a greater increase in FEV_1_ (9.4% vs 2.1%, *p* < 0.001) and forced vital capacity (FVC) (12.4% vs 7.3%, *p* < 0.01) compared to the control group. There were no differences in the number of days of antibiotic treatment, days of hospitalization or adverse events between the two groups [34].

A double-blind randomized placebo controlled trial compared the effect of DNAse administered once vs twice daily in 968 adults and children with CF treated for 24 weeks. The main outcome was the occurrence of respiratory exacerbations, reported in 27% of patients who took placebo, 22% and 19% of those treated with DNAse, once and twice daily, respectively. The administration of DNAse reduced the risk of respiratory exacerbation (by 28% and 37% respectively based on use for once or twice daily) and resulted in a significant increase in FEV_1_ vs placebo (on average + 5.8 and + 5.6%). The main reported side effects were alterations of the voice or laryngitis, but rarely severe and in any case resolving within 21 days of onset [31].

In 2004 Frederiksen et al. evaluated the effect of DNAse in reducing the number of bacterial infections of the lower respiratory tract in CF patients followed for one year. The number of positive cultures was significantly higher in the untreated group (82%) compared with the drug group (72%, *p* < 0.05), in particularly regarding the presence of *Staphilococcus aureus* (30% vs 16%, chi-squared test, *p* < 0.0001). Similarly, there was an increase in FEV_1_ of 7.3% in the treated group vs 0.9% in the placebo group [35]. In 2005 Robinson et al. evaluated the effect of DNAse on respiratory function and anatomical damage to the chest computed tomography (CT) scans in 25 children with CF treated for one year. After 3 months the group treated showed a reduction of 13% at T0 for the presence of air trapping compared to the untreated group that showed a 48% increase in the same parameter. After 12 months both groups showed a decline in FEV_1_ and FVC, but the treated group showed improvements in forced expiratory flow (FEF) 25–75, presence of air trapping, accumulations of mucus and scores on the chest CT scan. The authors concluded that the most sensitive outcome to evaluate the efficacy of DNAse in children with good respiratory function was the reduction over time of air trapping, compared to the parameters spirometry or chest CT scores [24]. In 2001 Quan JM et al. evaluated the long-term effect of DNAse (96 weeks) on spirometric parameters and exacerbations respiratory (239 children treated vs 235 placebo). The treated group showed a significant increase in FEV_1_ (32 ± 1.2%, p 0.006), FEF 25–75% (7.9 ± 2.3%, p 0.0008) and FVC (0.7 ± 1.0%, p 0.51). Furthermore, the risk of respiratory exacerbation was reduced by 34% in the treated group. There were no differences regarding the risk of adverse events in the two groups [36].

Paul K et al. evaluated the effect of DNAse on the inflammatory state at the lower respiratory tract in CF patients (105, aged ≥ 5 years), comparing the number of neutrophils and the concentrations of interleukin 8 in bronchoalveolar lavage. At the end of the period the treated group showed no increased inflammatory parameters in the broncho-alveolar lavage, differently to the control group [37]. In conclusion DNAse vs placebo comparison studies showed an increase in mean FEV_1_ of 9.51% (95% Cl 0.67–18.35) after one month of treatment (4 trials, 248 participants), 7.3% (95% Cl 4.04–10.56) after 3 months of treatment (1 trial, 320 participants, moderate quality evidence), 5.8% (95% Cl 3.99–7.61) after 6 months (1 trial, 647 participants, high quality evidence), 7.3% later one year of therapy (vs 0.9% in the placebo group, *p* < 0.005), 3.24% (95% Cl 1.03–5.45) after 2 years (1 trial, 410 participants) and finally a decline of—1.99% in the 3-year treated group vs -3.26% in the control group. There is no evidence to prove an improved quality of life in the treated group, on the other hand there is a reduction in the number of respiratory exacerbations after 2 years of treatment (RR 0.78, moderate quality evidence) [25].

In 2019 data from 4198 people in the UK CF Registry from 2007 to 2015 were evaluated in order to investigate the effects of one-, two-, three-, four and five-years of DNase use on lung function to see if the benefits of short-term treatment use are sustained over the long term. The treatment was estimated to be more beneficial in patients with lower lung function (FEV_1_ < 70%) using the drug for at least one year. These positive effects were maintained for up to 5 years of therapy [38].

Only one study was published comparing the effect of DNAse and mannitol on FEV_1_. Minasian et al. found a similar increase in FEV_1_ (6.7% vs 7.2%) in 38 children who were taking DNAse and mannitol for a period of 3 weeks. The combined use of the two drugs resulted in an increase FEV_1_ of 1.88% [39]. Recently a greater improvement in spirometric parameters was showed in a small cohort of CF children using DNAse and mannitol, compared to a control group using DNAse only [40].

We will not report the papers comparing DNAse and hypertonic saline, as already done by our group in a previous paper [41]. Anyway, the systematic reviews conclude that there is no superiority of hypertonic saline than other mucolytic agents [41].

### Adverse events

Regarding the risk of adverse events, 3 trials evaluated the percentage of episodes of haemoptysis in CF patients in DNAse therapy (in total 393 treated vs 395 controls) after one month and 6 months of therapy. There was no evidence of increased risk in the treated group (RR 0.88, 95% Cl 0.5–1.55). The risk of episodes of dyspnoea was no greater (4 trials for a total of 551 treated patientsvs 557 controls; RR 100.95% Cl 0.85–1.18) or pneumothorax (3 trials for a total of 393 patients treated vs 395 controls, RR 0.60, 95% Cl 0.08–4.5). The patients treated more frequently presented voice alterations (RR 1.69, 95% Cl 1.2–2.39) especially after the first 3 months of therapy or skin rash (RR 2.4.95% Cl 1.16–4.99). Rarely adverse events such as chest pain, cough, pharyngitis, conjunctivitis, wheezing, facial edema or changes in mucus [25].

### Effect of DNAse on sinonasal problems in Cystic Fibrosis patients

The benefit of DNAse in CF patients was also shown regarding sinonasal problems.

A prospective double-blind placebo-controlled cross-over trial conducted in 5 years and older CF individuals investigated the effects of sinonasal inhalation of DNAse delivered by vibrating aerosol pulses generated by PARI SINUS™. This form of drug administration significantly reduced sinonasal symptoms in the CF population in contrast to conventional inhalation that was not efficient enough to reach sinuses [42, 43]. Furthermore, Cimmino et al. revealed that children with CF receiving DNAse via sidestream nebulizer presented a significant improvement in sinonasal symptoms and Lund–Mackay scores [44]. Finally, a systematic review in 2018 on chronic rhinosinusitis in CF patients, confirmed these results [45].

### Effect of DNAse on rate of lung function decline in Cystic Fibrosis children

Recently a retrospective cohort study using the European CF Society Patient Registry evaluated whether lung function decline was impacted by chronic DNAse treatment. Analysis of patients < 18 years and with at least one year of data available before and after DNAse (*n* = 6065) showed a significant improvement in the rate of decline of lung function. The largest effect was observed in the < 12 years group, with an absolute difference in FEV_1_ rate of decline of 0.37% per year. There was no significant difference in lung function annual rate of decline per annum in the ≥ 18 years group [46].

### Effect of DNAse on lung clearance index in Cystic Fibrosis children

The LCI, measured by the multiple breath washout test, is a lung function outcome more sensitive than spirometry in correlating with airway changes seen on high-resolution computed tomography [47, 48]. It has been endorsed as a useful endpoint in clinical trials of patients with early or mild CF lung disease and as the main outcome measure in clinical trials with *CFTR* modulators in young people with CF [49–51]. A percentage change in LCI greater than ± 15% in preschool children can be considered physiologically relevant and greater than the biological variability of the test [52]. LCI reflects global ventilation inhomogeneity and is mainly influenced by small airway dysfunction [53]. Abnormal preschool LCI values were associated with concurrent measures of clinical status and later spirometry deficits [54].

Few studies have evaluated the effects of DNAse on LCI. Amin et al. in 2011 tested them on 17 CF patients aged 6 to 18 years and with FEV_1_ ≥ 80%. The drug was taken for 4 weeks followed by a washout period of 4 weeks. They show a significant improvement.

of LCI in the treated vs placebo group (0.90 ± 1.44; *p* = 0.022). There were no significant differences in value of FEV_1_, probably due to the good lung function of the patient group considered [55].

Recently, a single-centre, randomised, controlled, parallel group study evaluated the effects of one month’s withdrawal of nebulised DNAse in 5–18 years old children with CF. At the end of the month of withdrawal, an increase in the LCI value (1.74 (95% confidence interval: 0.62; 2.86) and a decrease in the FEV_1_ value (-6.8% predicted) were observed [56].

### A look towards the future: what is the perspective of treatments as DNAse for patients with Cystic Fibrosis treated with *CFTR* modulators?

For some years the introduction of new *CFTR* modulator drugs has transformed patients' lives with short- and long-term improvements in clinical outcomes. This can lead to changes in treatment patterns with less adherence in the group of patients taking these drugs. Hubert et al. found a decrease in prescribed DNAse two years after initiating ivacaftor and this was later confirmed in a larger population [57, 58]. It is unknown if changes in prescriptions resulted from physician or patient initiative. However, the differences observed between year 1 and year 2 suggest that DNase prescription modifications occurred after an extended period of time on ivacaftor once patient clinical symptoms improved [57].

However, to date conventional therapies are still indicated in CF patients treated with *CFTR* modulators. It will be interesting to extend this aspect when similar data become available from patients taking elexacaftor-tezacaftor-ivacaftor and provide precise indications to clinicians in order to reduce the treatment burden.

## Conclusions

To date DNAse is the only mucus degrading agent that has proven efficacy in CF. It reduces the number of pulmonary exacerbations and improves FEV_1_ and LCI in patients with CF. The single administration and the good tolerability make it highly available for prescription. Today, despite improvements in clinical parameters and survival in CF, an early use would be desirable in CF children from 6 years of age, especially in the presence of a pathological LCI value. The best benefits are shown in children < 12 years of age. In this group of patients DNAse slows the rate of decline in lung function. LCI is a useful marker to monitor early disease progression and guide the initiation of mucolytic therapy.

## Data Availability

Not applicable.

## Abbreviations

CF: Cystic Fibrosis
*CFTR*: Cystic Fibrosis Transmembrane Conductance Regulator
NBS: Newborn screening
DNAse: Dornase alfa
LCI: Lung clearance index
CFF: Cystic Fibrosis Foundation
FEV_1_: Forced Expiratory Volume in the 1st second
FVC: Forced vital capacity
CT: Computed tomography
FEF: Forced expiratory flow
PEP: Positive expiratory pressure
